# In Silico Study of Interactions between the Methylene Blue Molecule and the (TiO_2_)_20_ Cluster by Means of DFT Calculations

**DOI:** 10.1021/acsomega.4c00841

**Published:** 2024-06-17

**Authors:** Marco Antonio Meraz Melo, Alejandro Bautista Hernández, Mohammad Fereidooni, Christian Vianey Paz Lopez, Wilfredo Ibarra Hernandez, Odilon Vazquez-Cuchillo, Angel Pedro Rodríguez Victoria, Martin Salazar Villanueva

**Affiliations:** † Tecnológico Nacional de México/I.T. Puebla, Av. Tecnológico #420 Col. Maravillas, Puebla C.P. 72220, Puebla, México; ‡ Facultad de Ingeniería, Benemérita Universidad Autónoma de Puebla, Apdo. Postal J-39, Puebla 72570, Puebla, México; § Center of Excellence on Catalysis and Catalytic Reaction Engineering, Department of Chemical Engineering, Faculty of Engineering, Chulalongkorn University, Bangkok 10330, Thailand; ∥ Centro de Investigaciones en Dispositivos Semiconductores, Instituto de Ciencias, Benemérita Universidad Autónoma de Puebla, C.U., Puebla 72000, México

## Abstract

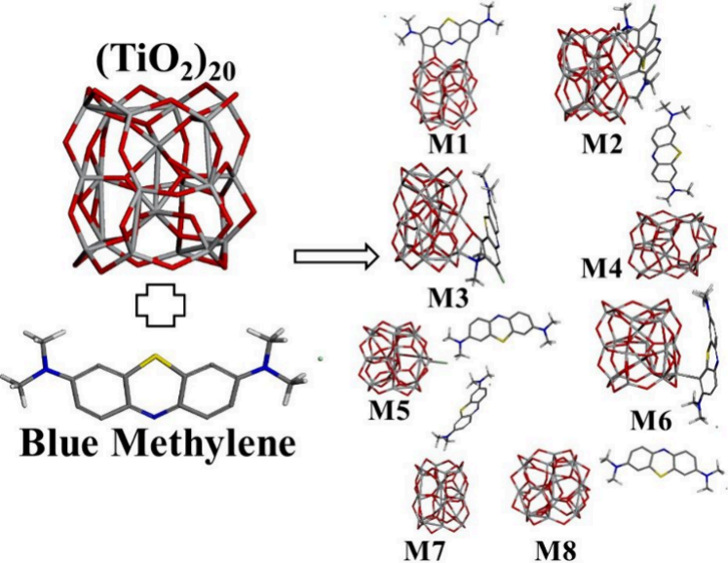

In this work, the (TiO_2_)_20_ cluster is proposed to adsorb the methylene blue (BM) dye; thus, the quantum parameters to explain the adsorption process are calculated by means of density functional theory calculations. Eight possible configurations are obtained and labeled from M1 to M8. According to the adsorption energy values, they reveal physisorption for at least two cases, and for the rest of the systems, they exhibit chemisorption. The preferential positions that lead to good adsorption for the BM dye are parallel to the semiconductor cluster; however, when one end of the BM dye formed by hydrogen atoms is interacting with the cluster, a weak chemical interaction is reached. The chemical interactions for M4 and M5 systems generate considerable increases of their electronic gap values (*E*_g_) with respect to the rest, and this effect is explained based on iso-surfaces of frontier orbitals and electronic charge transference. The chemical interactions between these chemical species are stable under vibrational and thermal criteria. This semiconductor cluster arises as a good candidate to adsorb some dyes like BM.

## 1 Introduction

Materials at the nanometric level exhibit unique properties compared to the bulk regime.^1−3^ In particular, semiconductor clusters such as those of titanium dioxide (TiO_2_)_*n*_ have been studied by experimental and theoretical scientific analyses.^4−6^ The low cost of TiO_2_, inertness, and nontoxicity make it a particularly good component for paints, ointments,^7^ food coloring,^8^ sunscreens,^9,10^ and many other industrial products. Due to the aforementioned, it is very important to understand the structure and growth pattern of these clusters for future scientific and technological applications; hence, Albaret et al. performed LSDA-DFT calculations to obtain structural and electronic properties at various sizes and stoichiometries for titanium dioxide clusters.^11^ Then, several groups of researchers have tried to elucidate the conformation of these (TiO_2_)_*n*_ clusters that opens the possibility to use them on several technological applications.^12−25^ According to the above investigations, these clusters have been studied for plenty of applications such as dye-sensitized solar cells,^26^ photocatalysis,^27^ optoelectronics,^28^ photoluminescence,^29^ catalysis,^30^ dissociative adsorption of water,^31^ and renewable energy^4^ or memristor.^32^ On the other hand, in order to establish the lowest energetic structures for medium sizes, the number of possibilities for different structures increases as size does; hence, the genetic algorithms (GA) are a very useful tool to find new structures. The above methodology has been implemented for titanium dioxide clusters successfully,^28,33−41^ and some of these structures have been synthesized by pulsed laser deposition.^42^ Our work group has reported new structures for (TiO_2_)_*n*_ (*n* = 15–20),^43^ and the upper sizes (*n* = 19 and 20) are good candidates to absorb some molecules present in textile processes such as MB, to help to remove this kind of harm molecule. In addition, in 1876, BM was first synthesized and later was recognized for its natural staining ability.^44^ BM has some medical applications, such as in methemoglobinemia treatment, and it has excellent antimalarial properties.^45−47^ BM is abundantly found in the textile industrial effluent, which can cause severe health problems for public and environmental ecology, and it consumes 1000 of the 100,000 types of dyes present in the commercial market.^48^ Methyl orange, rhodamine B, methylene blue (MB), Congo red, and Reactive Black-5 are classified as anionic, neutral, and cationic dyes; these are among the most widely used dyes in this industry.^49,50^ On the other hand, long exposition under a BM environment can cause significant health impacts such as anemia, cancer, eye irritation, nausea, vomiting, methemoglobinemia, and mental confusion. Thereby, the need to treat these dyes before discharging them to some affluent emerges; nevertheless, the first step is the conception of one device with the ability to sense or remove them. In this sense, different techniques have been applied to remove dyes from industrial wastewaters, including chemical oxidation, biological processes, flocculation, flotation, Fenton catalysis, electrocoagulation, membrane separation, and adsorption.^51−56^ Thus, zeolite surfaces have been used to adsorb this BM molecule, in which exothermic and spontaneous chemisorption was established.^57^ Several materials have been studied to remove the above molecule, such as bituminous coal,^58^ durable nanofibrous membranes,^59^ titanium dioxide,^60^ and zinc titanate^61^ surfaces, as well as TiO_2_ nanofibers^62^ and ZnO nanoparticles.^63^ On the other hand, by the theoretical side and prediction of possible materials (surfaces), Jaramillo-Fierro et al. have performed DFT calculation on surfaces of ZnTiO_3_ and TiO_2_(101), and they have found stronger adsorption the ZnTiO_3_ surface (−282.05 kJ/mol) than on TiO_2_ (−10.95 kJ/mol).^61^ Khnifira et al. carried out Monte Carlo simulations to determine the most stable low-energy configurations for the BM dye adsorption on the zeolite(001) face in an aqueous medium and in vacuum.^64^ Jaramillo-Fierro et al. have predicted a good value of adsorption energy of around 4.13 eV between BM and the anatase TiO_2_(101) surface; hence, this material arises as a good candidate to remove this dye.^65^ Al-Wasidi et al. performed effective removal of BM from aqueous solution using a metal–organic framework successfully.^66^ Thus, clusters have been studied to be applied as possible removers of this dye too; Oktavianti et al. have investigated zinc oxide (pristine and doped) nanocages to interact with this dye,^67^ and metal-decorated fullerenes have been studied within the DFT framework for the same aim.^68^ Partially summarizing, these efforts for removing BM molecules are due to their use in the textile industry and other dye applications; when they are discharged to water sources, they cause environmental pollution, and these are highly toxic to aquatic life, microorganisms, and human beings as they are mutagenic and carcinogenic.^69^ On the other hand, titanium dioxide clusters are good candidates to sense or remove these molecules because of their biocompatibility among other important properties; nevertheless, so far, investigations about titanium dioxide nanoparticles adsorbing BM molecules are focused on experimental investigations. The theoretical findings have been performed on surfaces and clusters as it has been already mentioned; however, it is very important for the adsorption behavior of titanium dioxide clusters at medium size to be compared to the bulk regime. Moreover, studies of AIMD (ab initio molecular dynamics) to analyze the PES (potential energy surface) and the evolution of the adsorption effect for semiconductor clusters interacting with harm molecules are still missing. Hence, this work addresses to analyze by means of ab initio calculations the largest semiconductor cluster of our previous work as a possible solution for this issue. The (TiO_2_)_20_ cluster has been chosen due to its geometry features previously reported by our work group^43^ to absorb in different positions to the BM molecule. This investigation is organized in this manner: in Section 2, computational parameters are given; in Section 3, the adsorption process is discussed by means of structural, thermal, and electronic properties, and then, some conclusions are drawn.

## 2 Computational Methodologies

The ground state of the whole set of titanium dioxide clusters bonded to BM molecules analyzed in this work was calculated by means of DMol^3^ software^70,71^ using DFT^72^ calculations with the subsequent parametrization. The generalized gradient approximation has been chosen to describe the exchange–correlation interaction, employing the Perdew–Burke–Ernzerhof (PBE) expression.^73^ To account for van der Waals forces, we used full optimization of the whole systems using the Tkatchenko–Scheffler (TS) scheme due to its ability to describe long large range interactions.^74^ We have selected a basis set composed of a double numerical basis (4s and 3d) with a polarized function (4p), and an all-electron calculation has been considered. The convergence criterion of optimization was set to 1 × 10^–5^ eV Å^–1^ for the energy gradient and 5 × 10^–4^ Å for the atomic displacements. The charge density is converged up to 1 × 10^–6^, which allows a total energy convergence of 1 × 10^–5^ eV. In the generation of the numerical basis sets, a global orbital cutoff of 5.2 Å was used. All calculations were carried out without spin restrictions, which allows for establishing the lowest-energy geometries. The condition of noncomplex frequencies was established as the stability criterion for the studied systems. The Mulliken analysis population was used to follow the electronic transference between these two chemical species.^75^ The electronic gaps (*E*_g_) were evaluated for the lowest-energy structures from their corresponding energy differences between the highest occupied molecular orbital (HOMO) and the lowest unoccupied molecular orbital (LUMO). It is well-known that values of the energy of the electronic gap (*E*_g_) are underestimated by using DFT calculations; however, in a previous report, they were validated for the (TiO_2_)_20_ cluster.^43^ Thus, with respect to the *E*_g_ value of BM, in the present work, a value of around 1.46 eV is obtained, which agrees with the experimental (1.91 eV)^76^ and theoretical (1.51 eV)^77^ values. On the other hand, vertical ionization potentials (VIPs) and vertical electron affinities (VEAs) have been calculated for each one of the eight models obtained in this study because based on Koopmans' theorem,^78^ these correspond to minus signs of HOMO and LUMO energies,^79^ respectively; hence, this is another way to obtain *E*_g_ values by means of ground electronic states. This quantum parameter can be calculated as the difference between VIPs and VEAs. Since VEAs are measured from the optimum structures, namely, from anionic semiconductor clusters (not from neutrals), this indicates that it is more quantitative to compare the calculated VIP, VEA, and VIP–VEA values to the occupied and unoccupied orbital energies and the *E*_g_ values than to compare their experimental values.^80^ In order to evaluate the thermal stability of two representative systems, ab initio molecular dynamics (AIMD) simulations with a canonical ensemble (NVT) at the temperature of 300 K are performed with a time step of 1 fs in 10 ps, respectively. We used the Vienna ab initio simulation package (VASP)^81,82^ with the generalized gradient approximation (GGA) of Perdew–Burke–Ernzerhof (PBE),^73^ for the exchange–correlation potential. The projector augmented wave (PAW) method was used for the description of the electron–ion interaction. The energy cutoff for the plane wave basis expansion was set to 600 eV. The *k*-point sampling uses the Monkhorst–Pack scheme and employs the gamma point. A supercell of 25 × 25 × 40 Å was adapted for the M3, M7, and M8 models. The adsorption energy (*E*_ads_) was calculated by means of the following expression:
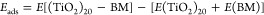
where *E*[(TiO_2_)_20_–BM], *E*(TiO_2_)_20_, and *E*(BM) are the total energies of the semiconductor cluster bonded to the BM molecule, the semiconductor cluster, and the BM molecule isolated, respectively. The basis set superposition error correction (BSSE) was not considered due to the nearly complete basis set for the separated atoms, which is implemented in DMol^3^ software.^83^ In this investigation, chemisorption is considered when one or more chemical bonds appear and physisorption for van der Waals interactions; namely, physisorption does not cause changes in the chemical bonding structure.^84,85^

## 3 Results and Discussion

### 3.1 Adsorption of the BM Molecule onto the (TiO_2_)_20_ Cluster

**Figure 1:**
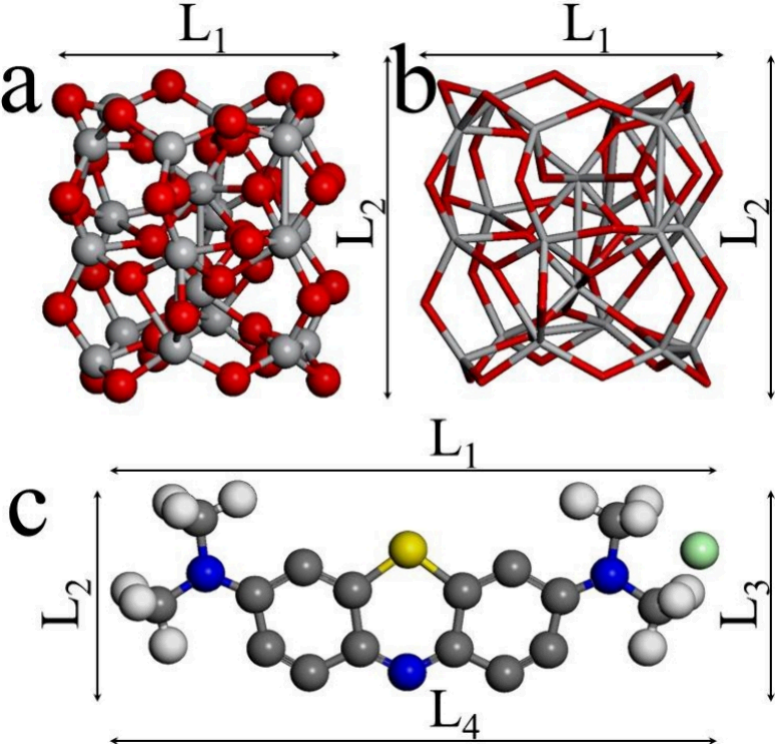
Models for the (TiO_2_)_20_ cluster and MB are depicted: (a) cluster with ball and sticks, (b) the same cluster with just sticks for better appreciation, and (c) MB dye. *L*_1_ and *L*_4_ and *L*_2_ and *L*_3_ labels mean the length and width of each model, respectively. Balls with color: red, gray, white, gray bold, blue, yellow, and cyan are related to oxygen, titanium, hydrogen, carbon, nitrogen, sulfur, and chlorine, respectively.

**Figure 2:**
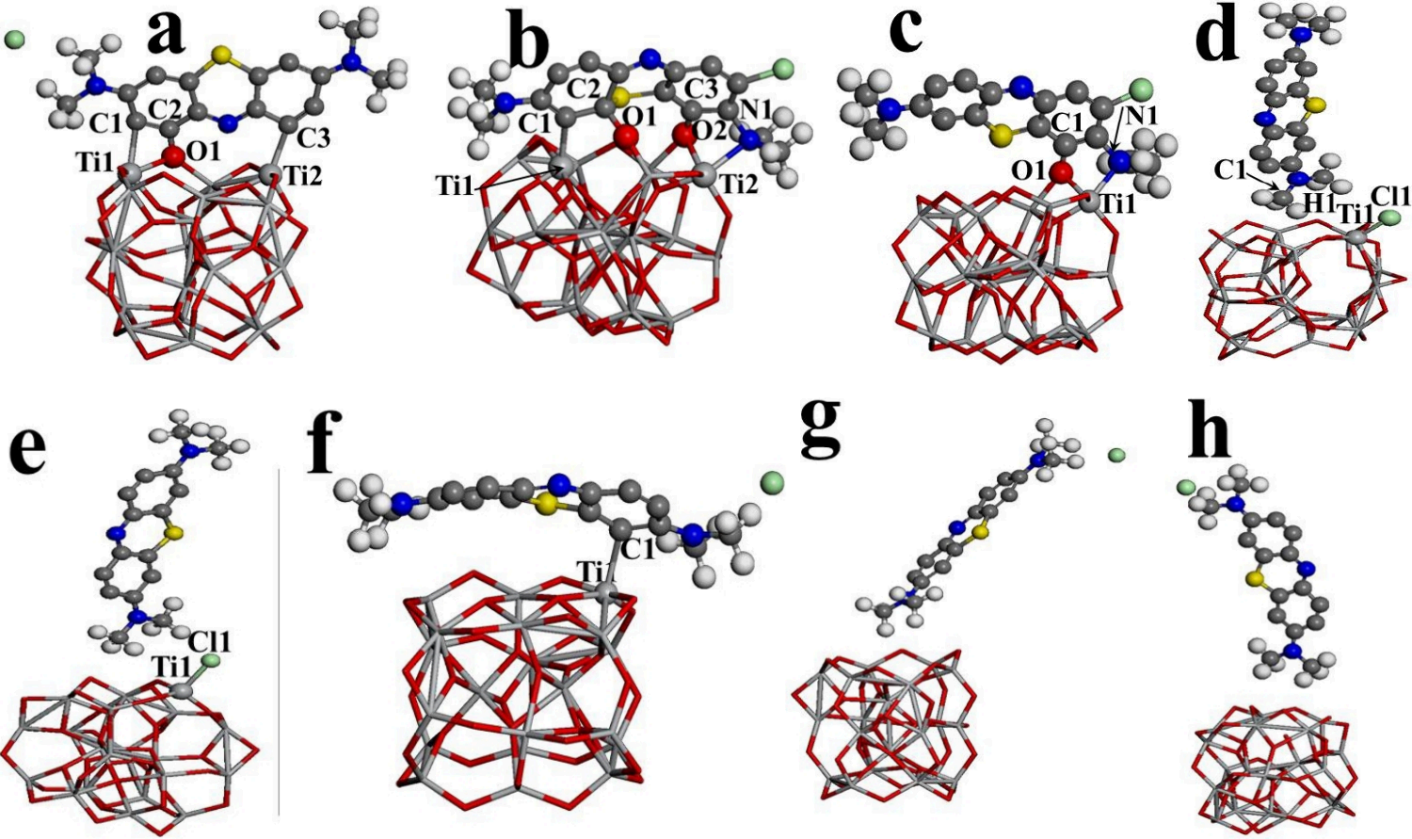
Representation of bonds for models from M1 up to M8 corresponding to labels from a to h.

The geometric structures of the (TiO_2_)_20_ cluster and BM molecule are depicted in Figure 1. The (TiO_2_)_20_ cluster has values of *L*_1_ = 6.66 Å and *L*_2_ = 7.61 Å, and BM possesses values of *L*_1_ = 13.89 Å and *L*_2_ = 4.15 Å. Thus, the *E*_g_ values for the titanium dioxide cluster and BM are 2.13 and 1.43 eV, respectively. It is expected that the values of *L*_1_ and *L*_2_ of the semiconductor cluster are shortened, lengthened, or bended due to the interaction with one BM molecule (see Figure 2). Hence, in order to cover the majority of possible structures generated by the BM–(TiO_2_)_20_ interaction and establish an adsorption tendency, several positions have been considered to interact between BM molecules and (TiO_2_)_20_ clusters, and due to the high symmetry of this semiconductor cluster, eight configurations or models were obtained after full optimization of the performance adsorption process between these two chemical species; these are depicted in Figure 2. For this purpose, these models are obtained among the interaction of axes *L*_1_ and *L*_2_ for the (TiO_2_)_10_ cluster and *L*_1_–*L*_4_ axes for BM, with both systems isolated.

**Figure 3:**
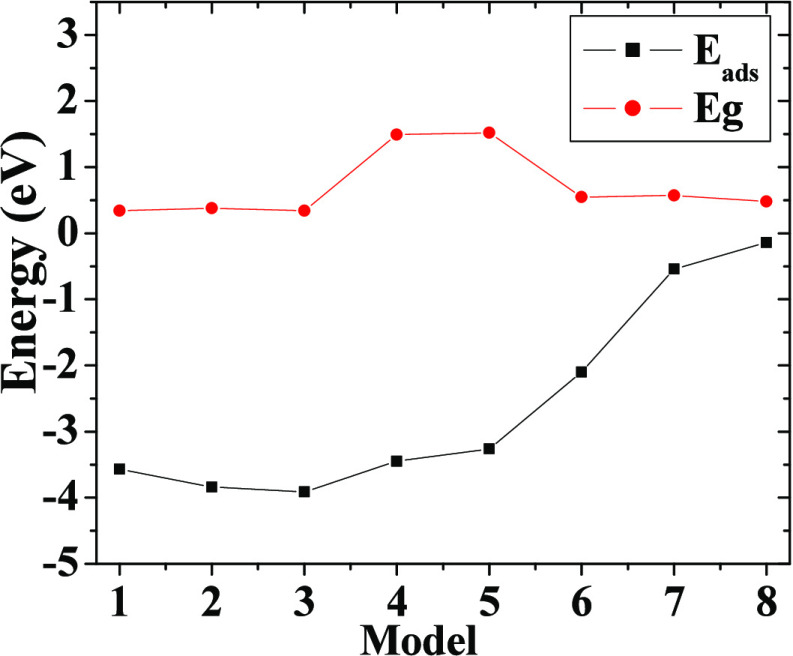
Adsorption energy (*E*_ads_) and gap energy (*E*_g_) are plotted, for the whole set of models considered.

These models are labeled as follows: model 1 (M1), model 2 (M2), and model 8 (M8). Their Cartesian coordinates are depicted in the Supporting Information to have better appreciation of these models (see Table 1S). Thus, in order to build M1, the BM molecule at *L*_1_ is located along *L*_1_ of the semiconductor cluster; after geometric optimization, three bonds appear, Ti1—C1, Ti2—C3, and O1—C2 with bond length values of 2.67, 2.25, and 1.35 Å, as is represented in Figure 2a, respectively. This effect is associated with electronic charge transference; this goes from titanium atoms of the cluster toward carbon atoms of the BM molecule, whereas the electronic charge of the carbon atom from the BM molecule migrates toward the oxygen atom of the cluster. Meanwhile, values of charge for carbon atoms at adsorption sites C1, C2, and C3 are −0.398, −0.751, and 0.340 e, respectively. The aforementioned leads to strong interaction; thus, a value of *E*_ads_ = −3.57 eV is generated, and this is related to chemisorption (see Table 1 and Figure 3). In this sense, M2 is obtained when the BM molecule is positioned along *L*_2_ of the semiconductor cluster and parallel to it, and then, the corresponding structure is generated and depicted in Figure 2b. Four bonds arise for this system: Ti1—C1, O1—C2, Ti2—N1, and O2—C3, whereas the following bond values are generated: 2.12, 1.41, 1.36, and 2.21 Å; these are displayed in the latter figure.

**Table 1:** Values of the Adsorption Energy (*E*_ads_), Frontier Orbitals (HOMO and LUMO), and Gap Energy^a^

	*E*_ads_	HOMO	LUMO	*E*_g_
M1	–3.57	5.95	5.61	0.34
M2	–3.84	5.21	4.83	0.38
M3	–3.91	5.17	4.83	0.34
M4	–3.45	6.40	4.91	1.49
M5	–3.26	6.36	4.85	1.52
M6	–2.10	6.20	5.65	0.55
M7	–0.54	5.75	5.19	0.57
M8	–0.14	5.69	5.21	0.48

a All of them are in eV.

The first three bonds follow the charge transference tendency related to Ti1, O1, and Ti2 atoms; their charge migrates toward atoms of BM molecules; unlike this process, the charge of the C3 atom goes toward the O2 atom of the semiconductor cluster. This effect generates a strong interaction between these chemical species; a value of *E*_ads_ = −3.84 e3 V is associated with chemisorption. However, the largest value of *E*_ads_ (−3.92 eV) is generated when the BM molecule is located along *L*_2_ of the semiconductor cluster and parallel to it, and this system (M3) is displayed in Figure 2c. This is similar to M2; however, M3 exhibits two bonds less than M2. Thus, two bonds are generated because of this chemical interaction as follows: O1—C1 and Ti1—N1; these have the corresponding bond length values of 1.30 and 2.21 Å, respectively. Despite having just two bonds between them, the rest of the MB molecules that are not bonded are close to the parallel surface of the titanium dioxide cluster (*L*_2_ direction), and apparently, this long-range interaction contributes to chemical adsorption. Moreover, the O1—C1 bond value is the shortest, and as a consequence, the electronic transference is stronger than the rest of the systems.

On the other hand, the M4 is generated when the BM molecule (*L*_3_) is placed on the semiconductor cluster in a perpendicular way, along *L*_1_. A Cl1—Ti1 bond is formed due to this chemical interaction, with a length of 2.29 Å. This effect is associated with electronic charge transference that goes from the titanium atom toward the chlorine atom; however, one hydrogen atom (H1) gives charge to the carbon atom (C1), as complementary charge transference (see Figure 2d). This bonding effect generates a value of *E*_ads_ = 3.45 eV, which falls into chemisorption as well. In a similar way to M4, the M5 model (Figure 2e) is generated by placing the BM molecule (*L*_3_) on the semiconductor cluster in a perpendicular way, but along *L*_2_. The bond Cl1—Ti1 has a length value of 2.27 Å, and charge transference is like the M4 case. M5 is 0.19 eV above the M4 case in adsorption energy; hence, these adsorption values are very close, and it does not matter which direction is chosen (*L*_1_ or *L*_2_) of the semiconductor cluster; the BM dye should be located perpendicular to (TiO_2_)_20_ to obtain the chemisorption effect, at least for M4 and M5 models. Thus, a value of *E*_ads_ = −2.10 eV is generated when the BM molecule by *L*_4_ is parallel to *L*_2_ of the semiconductor cluster (M6), whereas a Ti1—C1 bond is associated with a length of 2.29 Å; this effect is related to charge transference from the titanium atom (Ti1) toward the carbon atom (C1); despite this, *E*_ads_ is higher by 1.3 eV than the other models, and this process falls into chemisorption, too (see Figure 2f). Nevertheless, M7 and M8 models exhibit values of *E*_ads_ of around −0.54 and −0.14 eV, respectively. There are no bonds between the BM molecule and the titanium dioxide cluster for both models due to the fact that they have been constructed perpendicular to *L*_1_ and *L*_2_ directions of the (TiO_2_)_20_ cluster and the BM molecule from methyl ends (*L*_3_ and *L*_4_, respectively), as is shown in Figure 2g,h; hence, in these two cases, a physisorption process is generated. There is slight electronic charge transference from hydrogen atoms of the BM molecule toward the semiconductor cluster. On the other hand, according to coordination of titanium atoms at the adsorption zone, changes in *E*_ads_ values are exhibited; hence, in titanium oxide surfaces, the titanium atom possesses 6-fold coordination,^61^ whereas for the (TiO_2_)_20_ cluster, this variation is as follows: 4-fold coordination is associated with M1–M4, and 3-fold coordination and 5-fold coordination are related to M5 and M6, respectively. Electrostatic interactions are generated for M7 and M8, and these agree with a previous theoretical report.^61^ It is elucidated that the adsorption process governs for titanium atoms related to 3- and 4-fold coordination compared to 5-fold coordination, whereas in a previous report, for 6-fold coordination, *E*_ads_ values from 0.21 to 2.2 eV are obtained.^61,65^ From this point of view, it is important to obtain nanoparticles with low coordination on titanium atoms to obtain larger *E*_ads_ values.

### 3.2 Electronic Properties and Stability

**Figure 4:**
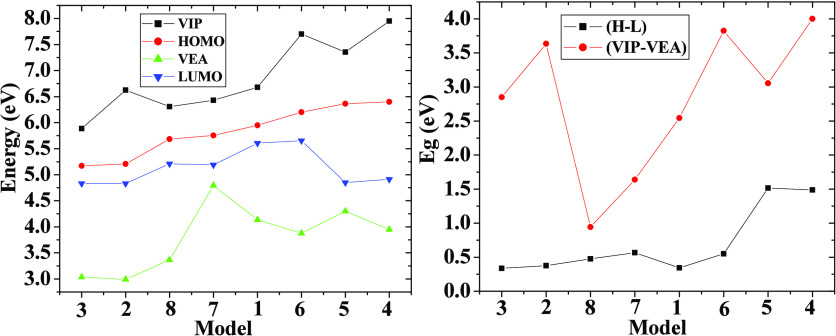
Plots for VIP, HOMO, VEA, and LUMO are depicted at the left side, whereas *E*_g_ plots from HOMO–LUMO (H-L) and VIP-VEA are depicted at the right side.

**Figure 5:**
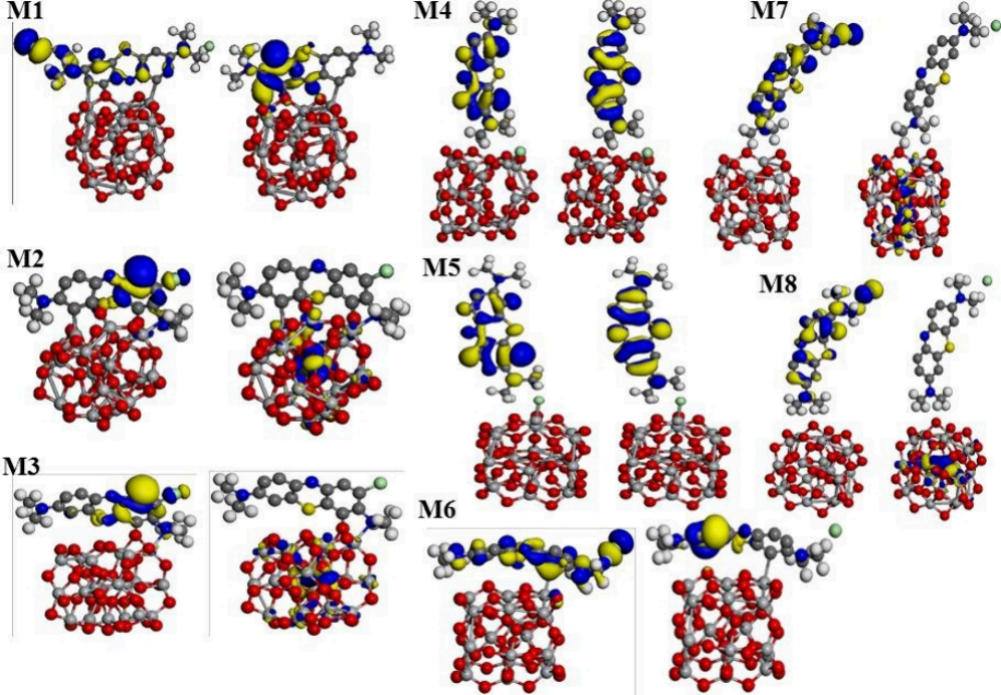
Eight models obtained in this study are depicted, from M1 to M8, respectively. The iso-surfaces of HOMO and LUMO are located at the left and right side of each model, and blue and yellow electronic densities are positive and negative, respectively. These are plotted at 0.03 eV/Å^3^.

The M1, M2, and M3 models exhibited values of the electronic gap that range from 0.34 to 0.38 eV, whereas for M6, M7, and M8 systems, electronic gap values range from 0.48 to 0.57 eV. Hence, when the BM dye is interacting from *L*_1_, *L*_2_, and *L*_4_ onto semiconductor clusters either from *L*_1_ or *L*_2_, these structural configurations or models fall within the range 0.34 ≤ *E*_g_ ≤ 0.57 eV. They are very active from the chemical point of view; moreover, these are prone to interacting with more BM molecules as well as with other kinds of harmful species. Nevertheless, for M4 and M5, there are associated values of 1.49 and 1.52 eV, respectively. Therefore, the interaction of the BM dye from *L*_3_ onto the semiconductor cluster either with *L*_1_ and *L*_2_ generates an increase of the *E*_g_ value due to the Cl1–Ti1 bonds exhibited in M4 and M5 contributing to the above electronic effect. Thus, for BM molecules anchored to a semiconductor cluster at these positions, for more than one molecule, they could not interact easily with the other ones. The above *E*_g_ values were calculated from energetic value differences between frontier orbitals (H-L); however, these should be compared to those obtained from VIP-VEA expression. From Figure 4, there is an acceptable tendency between values of (a) HOMO and VIP as well as (b) LUMO and VEA. Nevertheless, there are discrepancies between *E*_g_^H-L^ and *E*_g_^VIP-VEA^ values; this effect probably is generated by the functional chosen; a way to decrease this error is by means of GW calculations, and these values must be compared with a future experimental work to clarify this electronic issue. On the other hand, the iso-surfaces of the HOMO and LUMO indicate zones of low and high chemical reactivity, respectively; hence, the electronic distribution of the HOMO for M2 and M3 models is located at hexagons formed with one sulfur or nitrogen atom, and they exhibit σ and π—π bonds; meanwhile, for M1, in addition to electronic features, the methyl group at the left side has associated π*—π* bonds (see Figure 5). The electronic distribution of the LUMO for M2 and M3 models is located at the center of the semiconductor cluster, with the contribution of “d” electrons that come from the titanium atom; unlike for M1, this is placed at the first hexagon measured from left to right with the presence of σ bonds, principally. For M4 and M5 models, their HOMO and LUMO are displayed on the BM molecule with contributions of “s” and “p” electrons that come from carbon, nitrogen, and sulfur atoms; thereby, they exhibit π—π staking and σ bonds, resulting in the highest *E*_g_ values.

**Figure 6:**
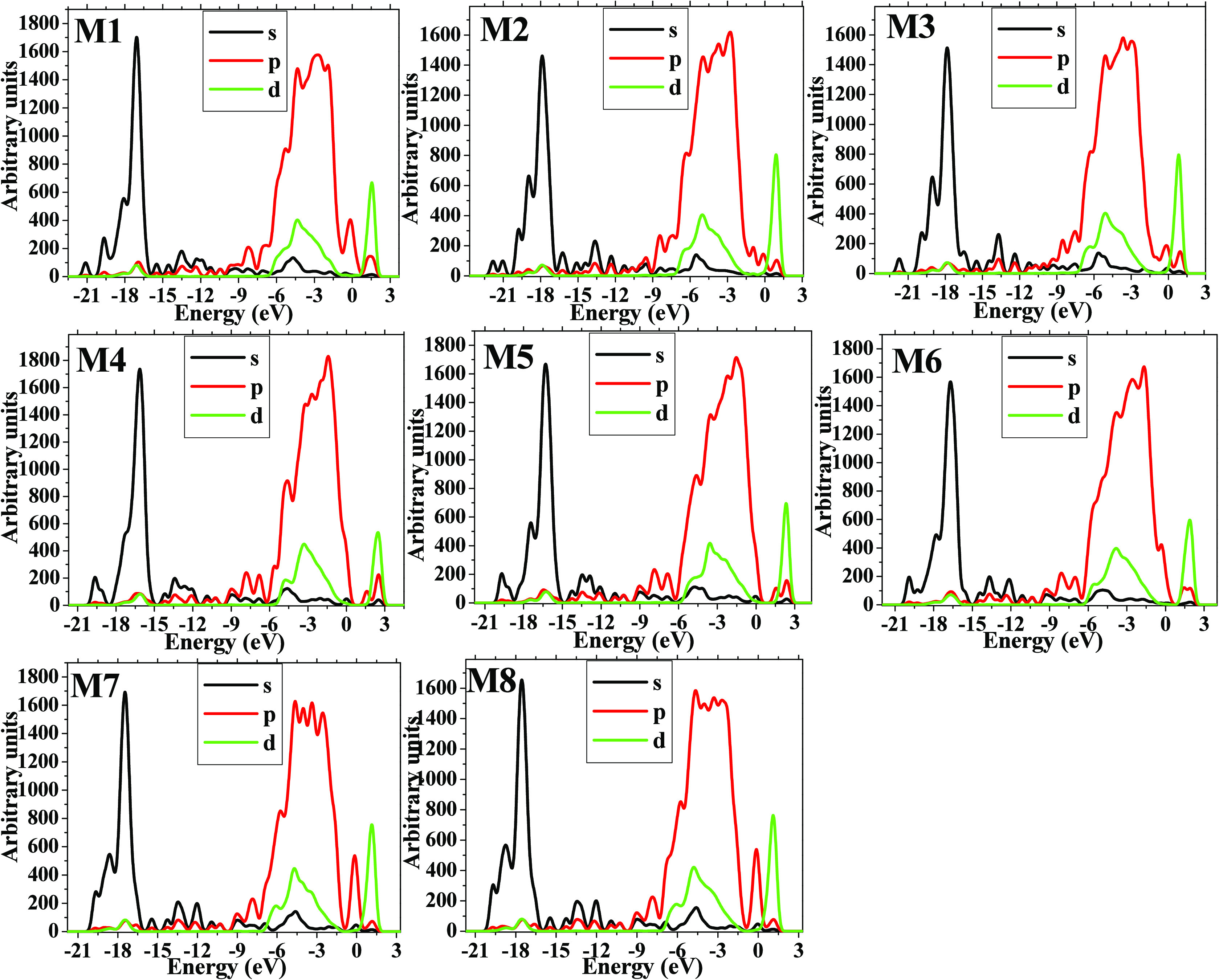
PDOS plots for the whole set of models are depicted.

For M6, M7, and M8, the electronic distribution of the HOMO is located on the BM molecule with a mixture of σ and π—π bonds; however, iso-surfaces of the LUMO for these systems are displayed at the center of them (“d” electrons); except for M6, this is found in a perpendicular way to HOMO distribution. This analysis is corroborated by PDOS plots of these models, where the left and right sides of the electronic gap correspond to HOMO and LUMO frontier orbitals, respectively (see Figure 6).

**Figure 7:**
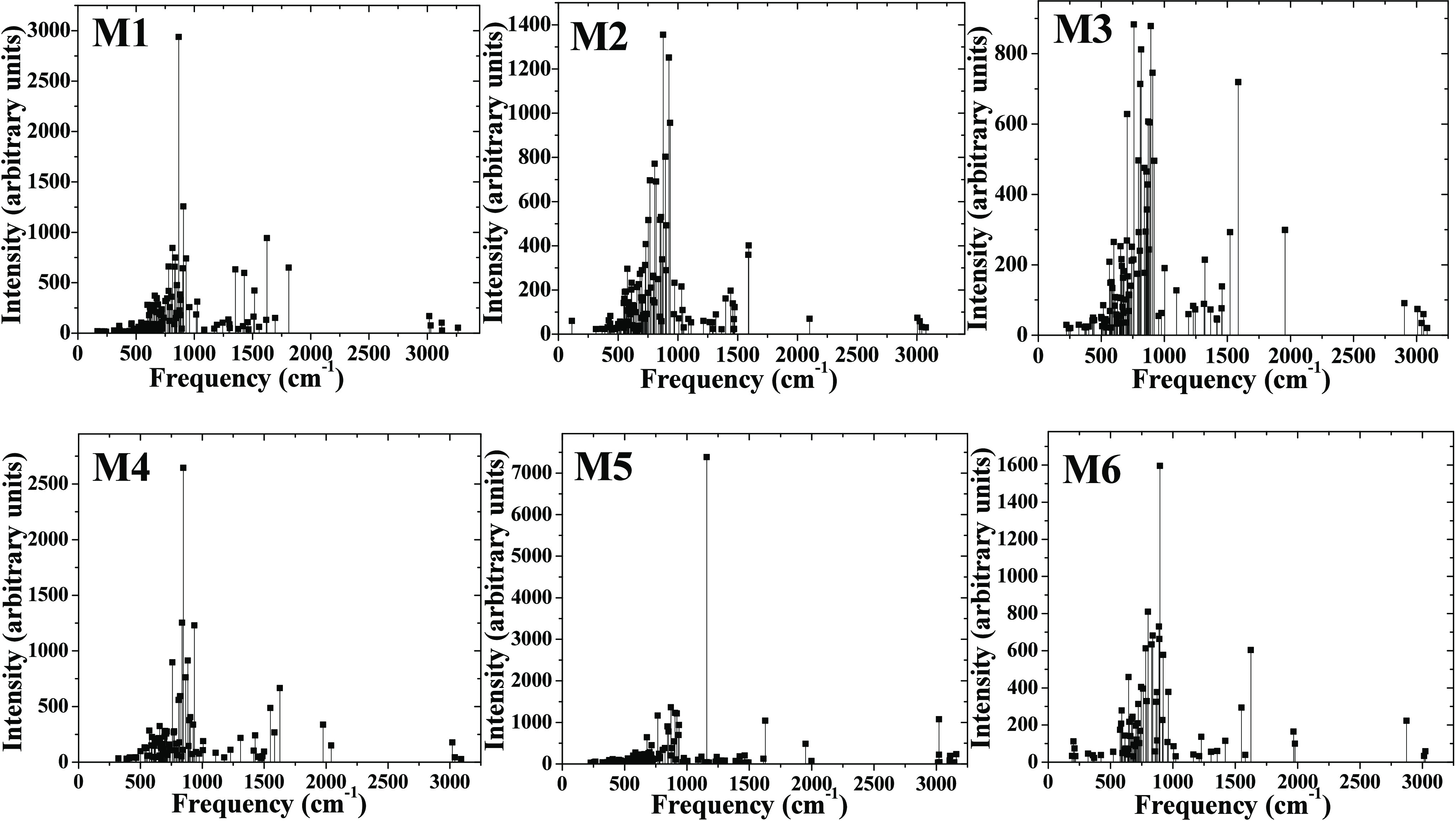
Vibrational spectra for M1–M6 models are depicted.

**Figure 8:**
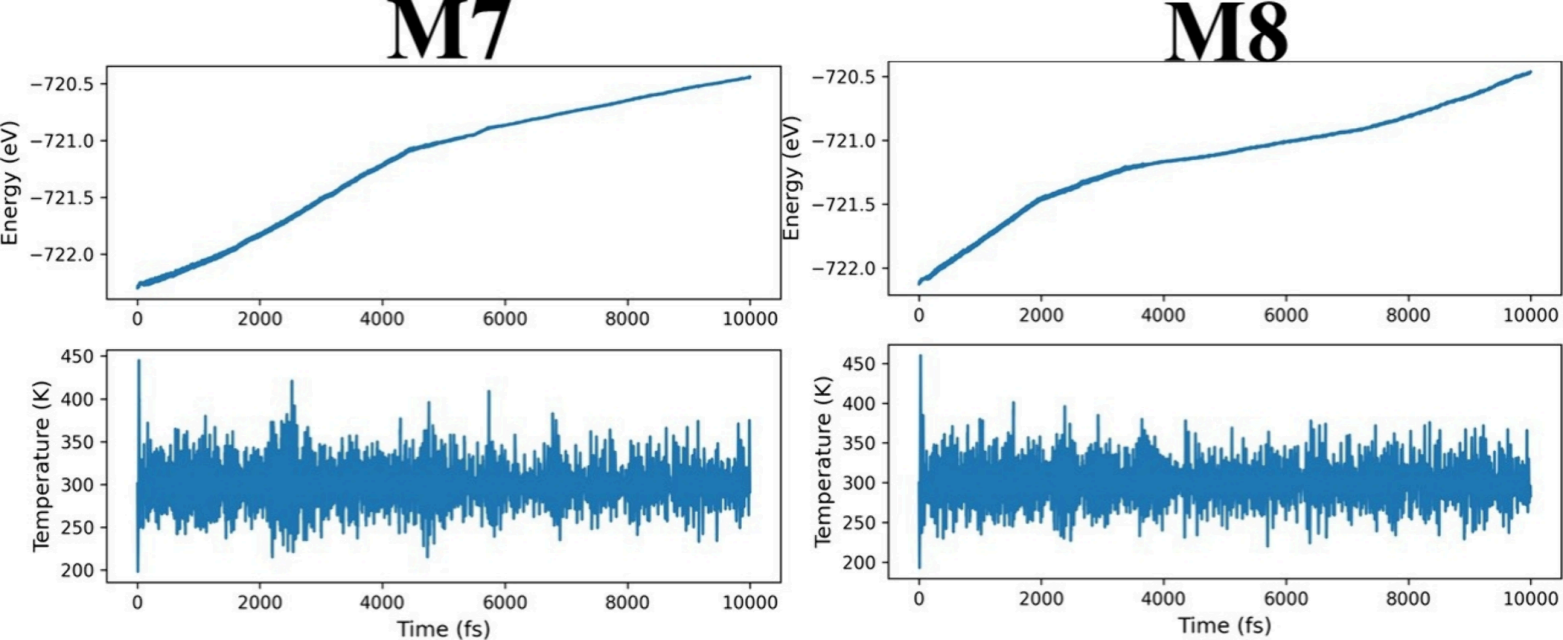
PES profiles for M7 and M8 models are depicted from 0 to 10.0 ps.

**Figure 9:**
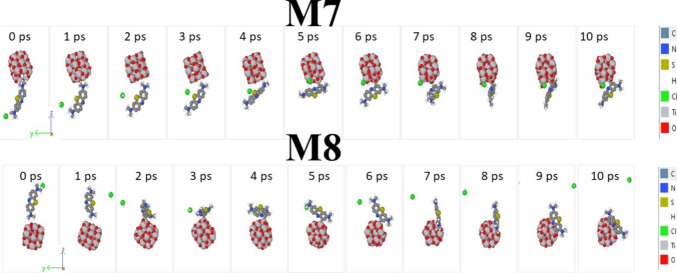
Snapshots of molecular dynamics trajectories for M7 and M8 at longer timescales (*T* = 300 K), with a time step of 1 fs.

Now focusing on vibrational stability, from M1 to M6 models, the vibrational modes related to the semiconductor cluster range from 300 to 950 cm^–1^, as well as a mix of stretching, scissors, and wagging modes arising from 955 to 2000 cm^–1^ for the BM molecule interacting with the (TiO_2_)_20_ cluster. The hydrogen atoms of the BM molecule exhibit stretching modes around 3000 cm^–1^. All of them exhibit real modes without imaginary modes, and vibrational spectra are depicted in Figure 7; therefore, these are stable vibrationally. In this sense, convergence criteria were not obtained to calculate vibrational spectra for M7 and M8; AIMD calculations were carried out to obtain PES plots during 10 ps, and from them, the thermal stability at 300 K can be elucidated for each one (see Figure 8) as well as the snapshots to follow possible considerable changes on their structures, as depicted in Figure 9. Thus, for M7, at the first 1000 fs, the chlorine atom is bonded to one hydrogen atom that comes from one methyl group to generate one molecule of HCl (hydrochloric acid), and in the next 3000 fs, this migrates toward the surface of the semiconductor cluster where it remains bonded up to 5000 ps. At 6000 fs, the HCl molecule is broken, the Cl atom is bonded to one titanium atom by 3-fold coordination, and the hydrogen atom is bonded to one oxygen; this last end has a OH radical form, and this is formed on one titanium atom with 4-fold coordination. These radical species play a key role in photocatalytic oxidation reactions.^76^ The above structural effect is associated with the change of the slope on the energetic plot from 4000 fs to 6000 ps. This structural configuration is retained for the rest of the range (next 4000 fs); the BM molecule suffers a structural restitution effect since at 8000 ps, this is oriented perpendicular to the (TiO_2_)_20_ cluster, and then, it is found in an inclined way with respect to *L*_1_ of the semiconductor cluster. On the other hand, for M8, from 0 to 2000 fs, the BM dye is located perpendicular to the semiconductor cluster and then almost parallel to it, whereas one HCL molecule is produced, and it begins to separate from the rest of the system. At 2000 fs, the first change of the slope appears on the PES plot; thus, up to 4000 fs, the BM dye is rotating under its self-axis as well as from the latter value up to nearly 8000 fs. The second change of the slope arises from 2000 to 4000 ps; from Figure 8, it is inferred that the BM dye tries to be orientated parallel to the (TiO_2_)_20_ cluster along *L*_2_, the HCl molecule moves away from the rest of M8, and this is located parallel to the *x*–*z* axis (coordinate axis from Figure 9). Finally, from the above value to 10,000 ps, the BM dye is oriented parallel to the *x*–*y* axis, and the HCl molecule practically has escaped from M8 due to having a distance between them of around 2 nm. It is seen that the ring formed of pure carbon atoms opposite to the chloronium atom is located at one titanium atom with 4-fold coordination. For M7 and M8, a degradation effect is found under AIMD calculations at room temperature (300 K) due to HCL being generated; this is bonded to the semiconductor cluster, and it is moving away from M7 and M8 systems. The possible production of HCL can help to create a repulsive effect by charge generation; moreover, this acidic environment prevents the particles from producing agglomerates, as it was pointed out by Maleki and Bertola.^76^ They proposed that pH should be maintained at around 4 to avoid recombination among titanium dioxide nanoparticles for sizes close to 4.5 nm. Thus, these AIMD calculations reveal that M7 produces OH radicals, and M8 can produce an acidic environment based on HCl molecules; the final structures are depicted in Figure S1 (see the Supporting Information).

## 4 Conclusions

DFT calculations were performed to evaluate the chemical interaction between the BM molecule and the (TiO_2_)_20_ cluster. From the M1 to M6 model, chemisorption is obtained; however, for M7 and M8 models, physisorption occurred due to weak interaction. Our findings reveal that the mechanism of electronic charge transference is from titanium and carbon atoms toward other chemical species, whereas for oxygen atoms, the process is inverse. The whole set of systems analyzed falls within the range 0.34 ≤ *E*_g_ ≤ 0.57 eV, except for M4 and M5, whose values were from 1.49 to 1.52 eV, respectively. The above effect indicates that when the BM molecule is oriented in a perpendicular way to the *L*_1_ or *L*_2_ direction of the semiconductor cluster, a high chemical stability is obtained. The whole set of models is vibrationally stable at least for M1–M6; nevertheless, for M7 and M8, AIMD calculations have been performed to explore their thermal stability. Hence, degradation of the BM molecule is exhibited for both cases, and a OH radical and a HCl molecule are produced as a consequence. These subproducts are desirable from an experimental point of view. Thereby, this titanium dioxide cluster of medium size can be used as a removal agent of textile dyes such as BM.

## Supporting Information Available

Final structures for M7 and M8 systems at 10 ps (300 K) and Cartesian coordinates of each of the models analyzed (PDF)
