# Supplementary material for: In Silico Study of Interactions between the Methylene Blue Molecule and the (TiO2)20 Cluster by Means of DFT Calculations
Source: ACS Omega. 2024 Jun 17;9(26):28018–27. doi: 10.1021/acsomega.4c00841 (PMC11223221; doi:10.1021/acsomega.4c00841)
Supplement: Supplementary file 1 — ao4c00841_si_001.pdf [file ao4c00841_si_001.pdf]

In-silico study of interactions between methylene blue molecule and $(TiO_2)_{20}$ cluster by means of DFT calculations

Marco Antonio Meraz Melo[1], Alejandro Bautista Hernández[2], Mohammad Fereidooni[3], Christian Vianey Paz Lopez[3], Wilfredo Ibarra Hernandez[2], Odilon Vazquez-Cuchillo[1], Angel Pedro Rodríguez Victoria[4] and Martin Salazar Villanueva[2*]

[1]*Tecnológico Nacional de México/I.T. Puebla, Av. Tecnológico #420 Col. Maravillas, C.P. 72220 Puebla, Puebla, México*

[2]*Benemérita Universidad Autónoma de Puebla, Facultad de Ingeniería, Apdo. Postal J-39, Puebla, Pue., 72570, México.*

[3]*Center of Excellence on Catalysis and Catalytic Reaction Engineering. Department of Chemical Engineering, Faculty of Engineering, Chulalongkorn University. Bangkok 10330. Thailand.*

[4]*Centro de Investigaciones en Dispositivos Semiconductores, Instituto de Ciencias. Benemérita Universidad Autónoma de Puebla. C.U, Puebla, México.*

*Corresponding author: martin.salazar@correo.buap.mx

Supporting information

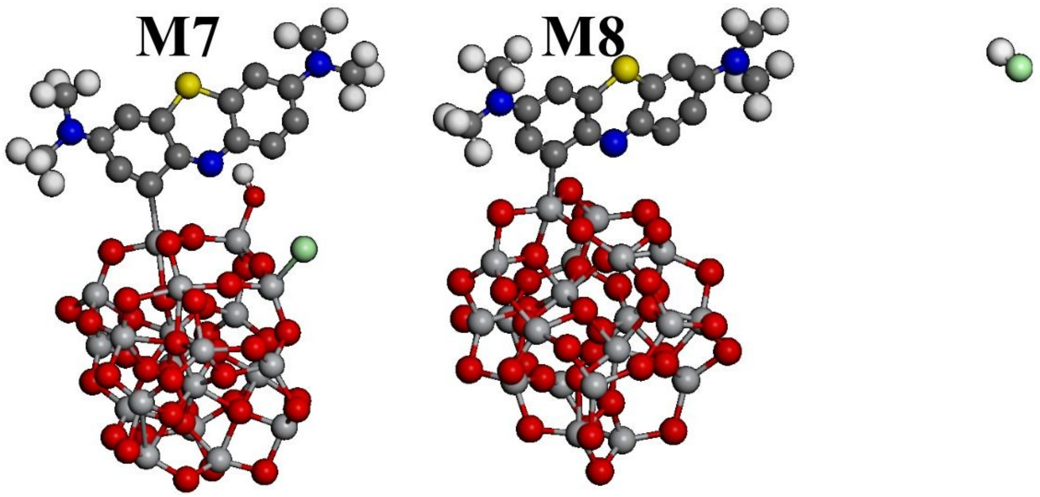

Figure 1S. Final structures for M7 and M8 systems at 10 ps (300K).

M1

	Atom	x	y	z
1	C	-0.636556	2.192235	-4.934798
2	C	-0.204493	2.264203	-3.534465
3	N	0.503779	1.340596	-2.911096
4	C	0.945207	0.221107	-3.435826
5	C	0.535054	-0.309359	-4.734461
6	S	-0.347025	0.722471	-5.861506
7	C	1.874185	-0.611243	-2.708126
8	C	2.147591	-1.913240	-3.061428
9	C	1.647259	-2.492676	-4.266409
10	C	0.891067	-1.579178	-5.013612
11	C	-1.662292	4.449822	-4.922383
12	C	-1.198893	4.330873	-3.614910
13	C	-0.575705	3.474048	-2.842607
14	C	-1.282745	3.241543	-5.505015
15	N	-2.306879	5.488806	-5.487190
16	N	1.823549	-3.777351	-4.602416
17	C	2.622283	-4.693917	-3.810478
18	C	1.192975	-4.344421	-5.779195
19	H	3.193851	-5.322499	-4.510965
20	H	3.297927	-4.137489	-3.159232
21	H	1.971177	-5.337606	-3.205398
22	H	0.343280	-4.972858	-5.483736
23	H	1.935432	-4.973229	-6.291705
24	H	0.837364	-3.534108	-6.422755

25	C	-2.721993	5.457807	-6.874758
26	H	-2.417171	4.506977	-7.317103
27	H	-2.253475	6.286379	-7.419983
28	H	-3.811558	5.566598	-6.939515
29	C	-2.621960	6.684424	-4.732534
30	H	-3.706623	6.846685	-4.726047
31	H	-2.141153	7.555758	-5.194641
32	H	-2.264357	6.573480	-3.706038
33	Cl	3.915497	-6.359761	-6.596690
34	Ti	-0.158619	-0.446990	1.194272
35	Ti	0.350282	-0.636185	4.024257
36	Ti	-0.200618	2.832948	-0.719440
37	Ti	-3.145306	1.442500	0.471106
38	Ti	-3.274519	-1.401329	0.139379
39	Ti	-1.062560	-3.611786	-0.196693
40	Ti	2.043173	-2.280436	-0.827694
41	Ti	2.286410	1.436669	-0.241537
42	Ti	0.785308	2.478627	5.585496
43	Ti	-1.915434	1.597703	5.868987
44	Ti	-2.301809	-1.590783	6.292774
45	Ti	-0.114902	-3.357170	5.711312
46	Ti	2.893183	-2.206816	4.832414
47	Ti	3.462766	0.571253	5.468106
48	Ti	3.173703	0.648999	2.324230
49	Ti	0.481421	2.622294	2.234480
50	Ti	-2.928866	2.117492	3.185850
51	Ti	-3.040599	-0.943904	3.255626
52	Ti	-0.158658	-3.965335	2.438704

53	Ti	2.680456	-2.853603	2.086658
54	O	-1.186591	-0.816844	2.877409
55	O	1.476924	-4.356388	1.943659
56	O	1.384659	-1.322366	0.641527
57	O	2.237950	2.265260	1.584117
58	O	-1.305873	1.931350	2.357732
59	O	-3.699363	0.521554	2.284403
60	O	3.858756	0.675567	0.679099
61	O	0.016435	3.774143	0.815624
62	O	-3.723188	2.800412	1.566634
63	O	-3.613091	-2.027567	1.815469
64	O	-1.081138	-4.916914	1.085923
65	O	3.256717	-3.054199	0.248868
66	O	4.014807	1.267704	3.905181
67	O	0.821524	3.307750	3.890407
68	O	-2.737568	2.957890	4.857851
69	O	-3.389778	-1.906430	4.882788
70	O	-0.599461	-4.321257	4.251333
71	O	3.524242	-3.467026	3.606387
72	O	-0.701134	-2.498760	1.205186
73	O	1.975476	-0.407496	4.970298
74	O	2.411717	1.869293	6.298451
75	O	-0.591262	2.853702	6.645525
76	O	-2.331228	0.107237	7.018492
77	O	-1.513084	-3.187958	6.942343
78	O	1.705741	-3.103242	5.984769
79	O	4.152666	-1.167836	5.743786
80	O	1.913740	2.873515	-1.110419

81	O	-1.956632	2.324695	-0.677440
82	O	-4.209948	0.117972	-0.372282
83	O	-2.606793	-2.811754	-0.872953
84	O	0.539392	-3.332927	-1.099731
85	O	2.372422	-0.238413	-1.508087
86	O	0.982084	0.462334	2.564508
87	O	-0.239427	1.020949	5.036549
88	O	-2.780118	0.589558	4.475382
89	O	-0.655319	-1.572885	5.366710
90	O	1.067316	-2.283420	3.382054
91	O	3.275900	-1.159554	2.744253
92	O	0.390267	1.139035	0.099207
93	O	-1.837150	-0.205710	0.347726

M2

1	C	-3.454322	-0.707776	-0.917973
2	C	-4.625071	-1.450762	-1.752233
3	N	-4.510645	-2.609363	-2.291325
4	C	-3.380920	-3.387254	-2.118659
5	C	-2.487400	-3.199766	-1.018084
6	S	-2.850491	-2.036437	0.263960
7	C	-3.064024	-4.372101	-3.050330
8	C	-1.860925	-4.993712	-3.101931
9	C	-0.832025	-4.582987	-2.227510
10	C	-1.213371	-3.785205	-1.103360
11	C	-5.011062	1.150949	-0.206070
12	C	-5.867632	0.517648	-1.177646
13	C	-5.730191	-0.572338	-1.757524

14	C	-3.765149	0.528492	-0.119692
15	N	-5.493994	2.202160	0.511603
16	N	0.607753	-4.704911	-2.401331
17	C	1.082458	-4.880585	-3.793822
18	C	1.289695	-5.673454	-1.510023
19	H	0.911769	-5.905181	-4.132465
20	H	0.560334	-4.188079	-4.457931
21	H	2.152395	-4.660322	-3.793575
22	H	2.366710	-5.525340	-1.617566
23	H	1.019651	-6.690748	-1.816038
24	H	1.002385	-5.495050	-0.474641
25	C	-4.848405	2.727709	1.693161
26	H	-4.232977	1.955687	2.156154
27	H	-5.631035	3.044865	2.392585
28	H	-4.214861	3.594553	1.467282
29	C	-6.643540	2.954961	0.048203
30	H	-6.332436	3.887526	-0.442873
31	H	-7.270226	3.207253	0.910557
32	H	-7.215947	2.355181	-0.665284
33	Cl	-1.645596	-6.429761	-4.138097
34	Ti	1.487697	0.626365	-0.624629
35	Ti	1.335080	0.978195	2.148687
36	Ti	2.887854	3.210950	-2.904597
37	Ti	-0.378550	2.725574	-2.718252
38	Ti	-1.043580	-0.029698	-2.936173
39	Ti	0.754235	-2.539578	-1.997687
40	Ti	3.984210	-1.638520	-1.565130
41	Ti	4.735818	1.365422	-1.796507

42	Ti	2.076021	4.231831	3.223724
43	Ti	-0.695277	3.960089	2.816572
44	Ti	-1.707638	0.960196	3.977087
45	Ti	0.099830	-1.230775	4.183490
46	Ti	3.384835	-0.762621	3.895712
47	Ti	4.223153	1.964514	4.147161
48	Ti	4.733867	1.351721	1.095404
49	Ti	2.741752	3.591859	0.028856
50	Ti	-0.805802	3.856537	-0.206672
51	Ti	-1.917032	1.269403	0.620812
52	Ti	0.653436	-2.287914	1.129655
53	Ti	3.629992	-1.938336	1.343742
54	O	0.043268	0.749724	0.747030
55	O	2.143968	-3.196295	1.018686
56	O	3.102468	-0.521878	-0.456888
57	O	4.435379	2.832407	-0.171416
58	O	0.880021	3.125130	-0.416385
59	O	-1.527543	2.194216	-1.000251
60	O	5.800111	0.816663	-0.252220
61	O	2.883796	4.475730	-1.629388
62	O	-0.945801	4.355772	-2.015689
63	O	-2.405958	-0.463068	-1.833281
64	O	-0.194487	-3.511559	-0.247097
65	O	4.682852	-2.519430	-0.111253
66	O	5.252095	2.225240	2.687487
67	O	2.786744	4.618220	1.549887
68	O	-0.725387	5.040121	1.267568
69	O	-2.393952	0.517829	2.434883

70	O	-0.242486	-2.367239	2.773773
71	O	3.903289	-2.387671	3.109569
72	O	0.720396	-1.240711	-0.624548
73	O	2.736348	1.117286	3.490549
74	O	3.282132	3.572509	4.458143
75	O	0.560870	5.070353	3.743605
76	O	-1.622996	2.827325	4.049164
77	O	-1.419168	-0.635269	5.037377
78	O	1.829934	-1.234407	4.842950
79	O	4.561315	0.272979	4.906236
80	O	4.694900	2.639348	-3.122251
81	O	1.205024	3.133982	-3.644772
82	O	-1.362207	1.553856	-3.821013
83	O	-0.111930	-1.614879	-3.327938
84	O	2.544126	-2.509563	-2.375806
85	O	4.824009	-0.317396	-2.566448
86	O	2.577490	1.626664	0.824554
87	O	0.878560	2.913607	2.582284
88	O	-1.778033	2.916388	1.356200
89	O	0.072901	0.549926	3.529337
90	O	1.844968	-0.876407	1.979295
91	O	4.357427	-0.275100	1.968975
92	O	2.784970	1.674978	-1.839078
93	O	0.349779	0.909553	-2.120602

M3

ATOM		X	Y	Z
1	C	-3.562605	-2.727452	1.414752

2	C	-4.828192	-2.766741	0.534597
3	N	-4.868043	-3.105128	-0.721697
4	C	-3.762528	-3.396581	-1.475078
5	C	-2.422420	-3.225775	-0.991738
6	S	-2.024324	-3.010041	0.677177
7	C	-3.914054	-3.817946	-2.802183
8	C	-2.873183	-3.939343	-3.660897
9	C	-1.594361	-3.458911	-3.288779
10	C	-1.369805	-3.173065	-1.925616
11	C	-4.707044	-2.122867	3.447155
12	C	-5.837651	-2.148730	2.580205
13	C	-5.943943	-2.407093	1.349056
14	C	-3.542520	-2.466689	2.745976
15	N	-4.753525	-1.798915	4.754904
16	N	-0.460326	-3.117182	-4.145640
17	C	-0.782048	-2.700933	-5.534224
18	C	0.590125	-4.168877	-4.163847
19	H	-1.013705	-3.572205	-6.151401
20	H	-1.632047	-2.017253	-5.529778
21	H	0.102050	-2.195320	-5.932818
22	H	1.435816	-3.789988	-4.742478
23	H	0.186987	-5.070253	-4.638617
24	H	0.911603	-4.386678	-3.144530
25	C	-3.553593	-1.782272	5.569547
26	H	-2.705487	-2.095018	4.955336
27	H	-3.671342	-2.466738	6.418673
28	H	-3.392371	-0.771268	5.966661
29	C	-5.985446	-1.422511	5.416121

30	H	-5.890095	-0.410592	5.829390
31	H	-6.193649	-2.115714	6.240299
32	H	-6.806910	-1.449909	4.696642
33	Cl	-3.097851	-4.813435	-5.201625
34	Ti	1.262323	1.512152	-1.298129
35	Ti	2.025634	1.187574	1.402295
36	Ti	1.546901	4.764195	-3.025183
37	Ti	-1.437481	3.862246	-1.843948
38	Ti	-1.962457	1.093128	-2.287645
39	Ti	0.086233	-1.248734	-3.104967
40	Ti	3.256749	-0.086679	-3.608752
41	Ti	3.772235	2.989697	-3.170491
42	Ti	2.943492	4.073028	3.098227
43	Ti	0.161014	3.487865	3.502190
44	Ti	-0.513132	0.310673	3.764836
45	Ti	1.476135	-1.630392	3.017315
46	Ti	4.504027	-0.715897	1.836194
47	Ti	5.360920	1.949652	2.545246
48	Ti	4.816193	2.270190	-0.570745
49	Ti	2.458119	4.407459	-0.190773
50	Ti	-0.942270	4.282069	0.915908
51	Ti	-1.486538	1.282363	0.849048
52	Ti	1.218319	-1.838613	-0.254951
53	Ti	4.012734	-1.088479	-0.935468
54	O	0.348675	1.146737	0.396069
55	O	2.582422	-2.476868	-1.117721
56	O	2.837855	0.571756	-1.979993
57	O	3.991870	3.936574	-1.190469

58	O	0.593277	3.919932	-0.019199
59	O	-2.023345	2.895488	0.029397
60	O	5.353206	2.244758	-2.289173
61	O	1.968967	5.655053	-1.532345
62	O	-1.706336	5.188881	-0.620492
63	O	-2.263315	0.375535	-0.649228
64	O	-0.103560	-2.765094	-1.597077
65	O	4.478078	-1.175692	-2.777120
66	O	5.801832	2.732802	0.977942
67	O	3.009116	4.980383	1.452398
68	O	-0.533919	4.994422	2.615608
69	O	-1.744078	0.239380	2.430955
70	O	0.908489	-2.464739	1.507506
71	O	4.841274	-1.954462	0.489841
72	O	0.601466	-0.396797	-1.510900
73	O	3.744561	1.120579	2.190957
74	O	4.546765	3.253086	3.609030
75	O	1.677839	4.520017	4.269059
76	O	-0.333792	1.955429	4.581061
77	O	0.133154	-1.387845	4.316971
78	O	3.324621	-1.537500	3.075103
79	O	5.939309	0.151127	2.650750
80	O	3.193114	4.524262	-3.987763
81	O	-0.285695	4.639351	-3.132750
82	O	-2.740174	2.728068	-2.631374
83	O	-1.344836	-0.208242	-3.522000
84	O	1.593251	-0.863329	-4.070513
85	O	3.644971	1.557879	-4.364000

86	O	2.670192	2.287571	-0.046204
87	O	1.703259	2.784887	2.527187
88	O	-0.914429	2.682609	2.123767
89	O	1.073499	0.193541	2.751404
90	O	2.506832	-0.516475	0.510139
91	O	4.824207	0.429726	-0.136861
92	O	1.915456	2.995049	-2.494947
93	O	-0.410739	2.090081	-2.020769

M4

1	Ti	0.001536	0.816439	2.297027
2	Ti	0.120730	1.055861	5.342920
3	Ti	1.015068	3.595631	0.784349
4	Ti	-2.223875	2.871108	0.732430
5	Ti	-2.889595	-0.158197	0.627846
6	Ti	-0.428415	-2.557438	1.449710
7	Ti	2.337002	-1.450416	1.661032
8	Ti	3.361300	1.195371	1.170774
9	Ti	0.248914	4.325996	6.564525
10	Ti	-2.464352	3.521721	6.062477
11	Ti	-3.291483	0.756472	6.581287
12	Ti	-1.361292	-1.285831	7.311101
13	Ti	1.854900	-0.543307	8.002907
14	Ti	2.606467	2.169479	7.636420
15	Ti	3.464932	1.895475	4.487284
16	Ti	1.580564	4.000546	3.589009
17	Ti	-3.659314	3.742433	2.994791

18	Ti	-3.885621	1.024763	3.759513
19	Ti	-0.118873	-2.443527	4.368103
20	Ti	2.565834	-1.529676	5.021412
21	O	-0.871061	0.711382	3.865028
22	O	1.381877	-3.178406	4.851283
23	O	1.850865	0.429648	1.936359
24	O	3.282504	3.812637	3.894430
25	O	-2.422197	2.475642	2.617759
26	O	-4.954041	2.443698	3.314108
27	O	4.203247	1.669089	2.674018
28	O	1.524601	4.922157	1.900823
29	O	-3.385785	4.280669	1.251992
30	O	-3.558414	-0.136328	2.293323
31	O	-0.830418	-3.506763	2.955710
32	O	3.054724	-2.037037	3.173416
33	O	3.827273	2.336391	6.317057
34	O	0.894750	5.006917	5.000252
35	O	-3.185686	4.487588	4.658068
36	O	-4.484429	0.138290	5.268864
37	O	-1.256024	-2.397500	5.871202
38	O	2.812574	-1.622804	6.914731
39	O	0.237382	-1.254981	2.582502
40	O	1.373252	0.942330	6.939300
41	O	1.517494	3.660004	7.777158
42	O	-1.436596	4.853317	6.986691
43	O	-3.403544	2.468813	7.312092
44	O	-3.062846	-0.785636	7.729891
45	O	0.179908	-1.204475	8.409050

46	O	2.851588	0.785853	8.878539
47	O	2.441817	2.527747	0.191778
48	O	-0.646524	3.586296	-0.042409
49	O	-3.183382	1.626902	-0.129865
50	O	-1.736211	-1.683442	0.531391
51	O	1.279709	-2.653874	0.687058
52	O	3.662643	-0.552364	0.629613
53	O	1.578409	2.066974	4.485683
54	O	-0.587635	2.835271	5.855635
55	O	-2.754317	1.830095	5.027239
56	O	-1.295081	0.373168	6.500865
57	O	0.886798	-0.736218	5.020078
58	O	3.468921	0.071752	4.782026
59	O	0.521275	2.695528	2.316393
60	O	-1.096282	0.855501	0.912666
61	C	0.109390	-2.567276	-8.801109
62	C	0.952018	-3.655839	-8.179234
63	N	1.333076	-3.688338	-6.915249
64	C	1.015641	-2.790426	-6.002396
65	C	0.093039	-1.617983	-6.222462
66	S	-0.587181	-1.328070	-7.798516
67	C	1.491068	-2.766823	-4.669471
68	C	1.169420	-1.890034	-3.827110
69	C	0.287713	-0.786101	-3.907428
70	C	-0.215040	-0.737652	-5.225560
71	C	0.294561	-3.461030	-11.081148
72	C	1.004217	-4.473795	-10.393696
73	C	1.299553	-4.598280	-9.173821

74	C	-0.174126	-2.532542	-10.136997
75	N	0.116889	-3.398807	-12.415197
76	N	0.001780	0.052197	-2.905985
77	C	0.618187	-0.105384	-1.604017
78	C	-1.002243	1.095350	-3.025697
79	H	-0.136000	-0.443446	-0.885967
80	H	1.427935	-0.834984	-1.652349
81	H	0.993698	0.868296	-1.272876
82	H	-0.617782	2.005723	-2.555818
83	H	-1.910145	0.797574	-2.486114
84	H	-1.239572	1.257957	-4.078888
85	C	-0.553209	-2.272078	-13.035765
86	H	-1.053685	-1.682911	-12.264455
87	H	-1.279102	-2.643873	-13.767196
88	H	0.178503	-1.641656	-13.557069
89	C	0.708336	-4.375159	-13.308701
90	H	1.535456	-3.921840	-13.870211
91	H	-0.050824	-4.716609	-14.021144
92	H	1.084833	-5.222321	-12.731413
93	Cl	-4.411972	-1.070575	-0.813656

M5

1	Ti	-1.408215	4.152403	-2.576416
2	Ti	-1.256704	4.189856	0.266960
3	Ti	-0.246598	7.016750	-4.430822
4	Ti	-3.519623	6.409884	-4.195845
5	Ti	-4.116701	3.294180	-4.349486

6	Ti	-2.110721	1.317892	-4.312814
7	Ti	0.953052	2.049358	-3.937882
8	Ti	1.895881	5.283111	-3.745786
9	Ti	-0.449472	7.220550	1.956832
10	Ti	-3.241541	6.462102	1.629168
11	Ti	-4.148739	3.372795	2.107651
12	Ti	-2.003484	1.477868	1.826253
13	Ti	0.914683	2.381688	1.765821
14	Ti	1.937455	5.053115	2.084831
15	Ti	2.744795	5.009588	-1.092514
16	Ti	-0.280117	7.161539	-1.324125
17	Ti	-3.837504	6.803691	-1.248131
18	Ti	-4.333557	3.952296	-1.040348
19	Ti	-1.727287	-0.271181	-1.155201
20	Ti	1.074790	1.685808	-1.068844
21	O	-2.355194	3.300688	-1.232280
22	O	-0.008548	0.028105	-1.142964
23	O	-0.105512	2.666964	-2.569215
24	O	1.503207	6.114856	-1.875104
25	O	-2.044697	7.457276	-1.221366
26	O	-5.236580	5.437472	-1.351647
27	O	3.516792	5.051077	-2.799629
28	O	-0.094969	8.060385	-2.981826
29	O	-4.315121	7.509663	-2.994659
30	O	-4.743195	3.045464	-2.697627
31	O	-2.383865	0.441945	-2.750124
32	O	1.910094	1.130348	-2.701142
33	O	3.099906	5.465663	0.719464

34	O	0.216363	7.812991	0.331015
35	O	-4.241894	7.479217	0.441530
36	O	-4.992684	3.142088	0.522488
37	O	-2.371866	0.268786	0.495941
38	O	1.857911	1.313655	0.610711
39	O	-2.232189	3.196109	-4.075238
40	O	0.562999	4.274142	1.173558
41	O	0.995384	6.479651	2.867634
42	O	-2.131843	7.788470	2.479979
43	O	-4.124670	5.162907	2.661838
44	O	-3.614657	1.714945	2.808664
45	O	-0.397765	1.430225	2.732567
46	O	2.145524	3.379324	2.831005
47	O	1.568672	6.663862	-4.906092
48	O	-2.010474	7.060303	-5.069575
49	O	-4.408353	4.980750	-5.057683
50	O	-3.751806	1.619556	-5.149842
51	O	-0.390180	1.170664	-4.955831
52	O	1.744030	3.586746	-4.540822
53	O	-0.648889	5.208882	-1.147358
54	O	-1.384578	5.722069	1.505104
55	O	-3.134349	5.263403	-0.025917
56	O	-2.380093	3.320914	1.571565
57	O	-0.447483	2.351807	0.257800
58	O	2.005830	3.390214	-1.082990
59	O	-0.203039	5.338987	-3.702221
60	O	-2.879545	5.401513	-2.800518
61	C	2.563179	-9.166167	2.492073

62	C	2.372189	-8.709641	3.919803
63	N	2.094965	-7.472225	4.291745
64	C	1.947971	-6.447411	3.473433
65	C	2.102241	-6.502986	1.976004
66	S	2.489462	-8.010339	1.193191
67	C	1.626595	-5.122834	3.859708
68	C	1.484167	-4.171403	3.048678
69	C	1.601617	-4.107885	1.639676
70	C	1.957703	-5.397931	1.185728
71	C	2.907796	-11.526908	3.085927
72	C	2.749517	-10.994038	4.386912
73	C	2.521947	-9.818267	4.784108
74	C	2.810093	-10.469921	2.164826
75	N	3.108984	-12.826813	2.792003
76	N	1.410320	-3.018105	0.888922
77	C	0.949635	-1.771683	1.474664
78	C	1.544367	-3.041427	-0.556771
79	H	-0.111604	-1.635499	1.236438
80	H	1.082607	-1.793669	2.557717
81	H	1.499233	-0.936266	1.031490
82	H	2.157975	-2.188832	-0.863194
83	H	0.551877	-2.921356	-1.009401
84	H	1.990204	-3.987909	-0.867030
85	C	3.209531	-13.289405	1.421010
86	H	3.193925	-12.428851	0.749517
87	H	2.367153	-13.954118	1.191982
88	H	4.141810	-13.852125	1.293674
89	C	3.174027	-13.843723	3.822607

90	H	4.100111	-14.419102	3.706798
91	H	2.323681	-14.530510	3.726407
92	H	3.152938	-13.373112	4.807618
93	Cl	-2.009281	-2.524923	-1.244757

M6

1	C	-4.470657	0.838730	0.864417
2	C	-4.830874	2.194592	0.403853
3	N	-5.043199	2.543567	-0.858711
4	C	-4.741783	1.773249	-1.886155
5	C	-4.056431	0.441074	-1.806639
6	S	-4.166922	-0.425683	-0.291244
7	C	-4.891147	2.119318	-3.251336
8	C	-4.350181	1.510931	-4.198753
9	C	-3.659356	0.276593	-4.205644
10	C	-3.396050	-0.191501	-2.856432
11	C	-4.362863	1.435532	3.299280
12	C	-4.693621	2.692173	2.749797
13	C	-4.920612	3.014716	1.551229
14	C	-4.347095	0.574728	2.198579
15	N	-4.113538	1.122853	4.583062
16	N	-3.270068	-0.347518	-5.313711
17	C	-3.410720	0.259152	-6.622932
18	C	-2.798115	-1.717885	-5.342865
19	H	-4.030292	-0.412999	-7.240092
20	H	-3.856560	1.250823	-6.532872
21	H	-2.418046	0.329118	-7.081844

22	H	-1.753952	-1.740538	-5.672131
23	H	-3.416510	-2.244097	-6.093761
24	H	-2.895786	-2.181191	-4.363209
25	C	-3.820892	-0.238568	4.983426
26	H	-3.836530	-0.895915	4.110752
27	H	-4.565552	-0.585676	5.711178
28	H	-2.836944	-0.261903	5.468618
29	C	-3.956281	2.141620	5.603194
30	H	-2.906605	2.173780	5.922472
31	H	-4.582385	1.897338	6.468648
32	H	-4.249197	3.114133	5.202945
33	Cl	-4.950061	-2.565133	-7.859329
34	Ti	2.042143	-0.113167	-1.061200
35	Ti	2.324947	-0.599248	1.798306
36	Ti	3.270109	2.731009	-2.898049
37	Ti	0.046497	2.049329	-2.464276
38	Ti	-1.184010	-0.552738	-2.398807
39	Ti	0.901705	-3.102646	-2.514529
40	Ti	4.101425	-2.573421	-2.791993
41	Ti	4.860604	0.217479	-2.773947
42	Ti	2.883799	2.484071	3.319185
43	Ti	0.113417	2.072649	3.125375
44	Ti	-0.738892	-1.010917	3.687108
45	Ti	1.035382	-3.193659	3.567676
46	Ti	4.366436	-2.642968	3.614509
47	Ti	5.279326	0.075518	3.661869
48	Ti	5.439530	0.299671	0.370561
49	Ti	3.427958	2.617572	0.015956

50	Ti	-0.246654	2.542981	0.268302
51	Ti	-0.996884	-0.404607	0.553430
52	Ti	1.483185	-3.737149	0.231956
53	Ti	4.400114	-3.175998	0.479925
54	O	0.885821	-0.524542	0.545877
55	O	2.988589	-4.598545	0.194107
56	O	3.524993	-1.005690	-1.968734
57	O	5.139571	2.172529	0.271177
58	O	1.409211	1.961027	-0.326986
59	O	-1.069412	0.934030	-0.836101
60	O	6.055878	0.304934	-1.409573
61	O	3.522089	3.810467	-1.413436
62	O	-0.655497	3.376839	-1.344453
63	O	-1.529607	-1.508202	-0.816821
64	O	0.554647	-4.414663	-1.301757
65	O	4.808467	-3.461607	-1.391399
66	O	6.188505	0.353663	2.151798
67	O	3.153441	3.335358	1.752159
68	O	-0.067477	3.474176	1.874059
69	O	-1.655580	-1.241156	2.170631
70	O	0.693673	-3.972151	1.984538
71	O	5.016218	-3.610929	2.236943
72	O	1.546721	-2.175104	-1.094494
73	O	3.792242	-0.904382	3.048582
74	O	4.329125	1.579877	4.149654
75	O	1.396438	3.055224	4.175064
76	O	-0.720301	0.742886	4.260684
77	O	-0.487342	-2.688618	4.517365

78	O	2.765773	-3.331586	4.266878
79	O	5.609122	-1.591649	4.515157
80	O	4.606302	1.787236	-3.801948
81	O	1.535507	2.720705	-3.431479
82	O	-1.148876	1.172672	-3.455621
83	O	-0.471441	-2.017770	-3.246898
84	O	2.501804	-3.262848	-3.460886
85	O	5.196449	-1.376508	-3.714146
86	O	3.388975	0.384641	0.597272
87	O	1.734248	1.144107	2.643828
88	O	-0.925165	1.248288	1.609466
89	O	1.028397	-1.358646	3.108820
90	O	2.755931	-2.315443	1.124310
91	O	5.192603	-1.545451	0.436558
92	O	3.346532	1.301703	-1.722849
93	O	0.852111	0.165953	-2.332494

M7

1	C	-1.629611	-0.097250	-6.005912
2	C	-0.495408	-0.915417	-6.585688
3	N	-0.101174	-0.883319	-7.846505
4	C	-0.640623	-0.137270	-8.797042
5	C	-1.787574	0.817087	-8.599147
6	S	-2.497550	1.015202	-7.021331
7	C	-0.245494	-0.111343	-10.157428
8	C	-0.799320	0.605188	-11.034853
9	C	-1.872379	1.526094	-10.949940

10	C	-2.327616	1.555398	-9.616062
11	C	-1.449592	-1.056492	-3.753900
12	C	-0.378066	-1.743144	-4.375710
13	C	0.060780	-1.717032	-5.559815
14	C	-2.014009	-0.183226	-4.697091
15	N	-1.860712	-1.222414	-2.479946
16	N	-2.388563	2.231518	-11.971040
17	C	-1.825707	2.197746	-13.300958
18	C	-3.439327	3.206354	-11.780320
19	H	-1.533285	3.234019	-13.563899
20	H	-0.972400	1.518794	-13.335939
21	H	-2.597175	1.887625	-14.015908
22	H	-4.289342	2.953387	-12.425040
23	H	-3.042792	4.183491	-12.119476
24	H	-3.736224	3.231205	-10.730930
25	C	-3.004379	-0.500633	-1.954460
26	H	-3.223008	0.348543	-2.605278
27	H	-2.775577	-0.161943	-0.938492
28	H	-3.882708	-1.158473	-1.911490
29	C	-1.308140	-2.252433	-1.624159
30	H	-2.002656	-3.099281	-1.541891
31	H	-1.157433	-1.838485	-0.622963
32	H	-0.354210	-2.597645	-2.025391
33	Cl	-1.816430	5.599090	-13.433405
34	Ti	0.408673	-0.779863	2.913552
35	Ti	1.837536	-0.101051	5.299652
36	Ti	-0.470260	1.891443	0.619586
37	Ti	-2.811987	0.893399	2.829635

38	Ti	-2.759197	-1.984550	3.134257
39	Ti	-0.534872	-4.153754	2.450367
40	Ti	2.044326	-2.856281	0.656039
41	Ti	1.705726	0.206761	-0.163169
42	Ti	2.418397	3.346177	6.085699
43	Ti	0.094623	2.313725	7.388176
44	Ti	0.124818	-0.676314	8.440015
45	Ti	2.186531	-2.452559	7.555013
46	Ti	4.632111	-1.381113	5.679868
47	Ti	5.131450	1.468570	5.238577
48	Ti	3.644646	1.000999	2.411676
49	Ti	1.119092	2.759943	3.252901
50	Ti	-1.817075	2.139788	5.175034
51	Ti	-1.568209	-0.816460	5.828052
52	Ti	1.234803	-3.873670	4.635825
53	Ti	3.679913	-2.513652	3.117125
54	O	0.068327	-0.738688	4.816582
55	O	2.702216	-4.138203	3.759402
56	O	1.811316	-1.730907	2.062770
57	O	2.776961	2.651408	2.559144
58	O	-0.566467	1.921492	3.901513
59	O	-2.683407	0.384949	4.938135
60	O	3.248988	0.795843	0.575074
61	O	0.230323	3.195715	1.637916
62	O	-3.170446	2.401570	3.754368
63	O	-2.454553	-2.220523	4.899685
64	O	0.088019	-5.163148	3.845173
65	O	3.670974	-3.263167	1.331991

66	O	5.134559	1.641472	3.454983
67	O	1.554287	3.977466	4.562374
68	O	-1.214610	3.321157	6.529743
69	O	-1.330323	-1.399254	7.616672
70	O	1.414112	-3.731288	6.528007
71	O	5.050494	-2.609226	4.393654
72	O	0.120572	-2.749999	3.453538
73	O	3.669512	0.354206	5.447710
74	O	4.242408	2.902811	6.064821
75	O	1.451235	3.697518	7.555896
76	O	0.167222	1.115039	8.880259
77	O	1.210392	-2.067926	9.100978
78	O	3.988856	-2.110945	7.275552
79	O	5.998618	-0.093754	5.834616
80	O	0.563751	1.463888	-0.912156
81	O	-2.177277	1.479272	1.118933
82	O	-3.943745	-0.620447	2.692621
83	O	-2.293066	-3.552797	2.238821
84	O	0.569303	-3.977927	0.973226
85	O	1.813598	-1.560876	-0.689365
86	O	1.759721	0.602034	3.464833
87	O	1.500451	1.718946	6.069386
88	O	-0.952323	0.915992	6.532204
89	O	1.422233	-0.784392	7.061413
90	O	2.589627	-1.785674	4.828863
91	O	4.083641	-0.750460	2.769294
92	O	0.429650	0.348301	1.260241
93	O	-1.458492	-0.705956	2.644656

M8

1	Ti	-1.198192	4.200159	-2.087893
2	Ti	-1.047795	4.152046	0.774932
3	Ti	-0.428290	7.205009	-4.056669
4	Ti	-3.645088	6.366343	-3.560747
5	Ti	-4.126492	3.549352	-3.710213
6	Ti	-2.010709	1.184560	-3.668146
7	Ti	1.226055	2.294022	-3.609649
8	Ti	1.724023	5.423267	-3.447166
9	Ti	-0.601840	7.276174	2.257690
10	Ti	-3.386523	6.677991	2.027741
11	Ti	-4.128576	3.483971	2.548792
12	Ti	-1.969789	1.576698	2.218044
13	Ti	1.166492	2.387329	1.983258
14	Ti	1.870430	5.146903	2.601668
15	Ti	2.080293	5.066104	-0.584961
16	Ti	-0.242719	7.213367	-1.062142
17	Ti	-3.794248	7.163097	-0.846439
18	Ti	-4.339718	4.168670	-0.616155
19	Ti	-1.621177	0.773302	-0.894759
20	Ti	1.364562	1.659381	-0.743641
21	O	-2.452906	4.059253	-0.622421
22	O	0.060022	0.285833	-1.074759
23	O	0.434357	3.137868	-2.196741
24	O	1.481562	6.636940	-1.568452
25	O	-2.081459	6.693242	-1.321159
26	O	-4.573282	5.627817	-1.817434

27	O	3.000375	4.801550	-2.112997
28	O	-0.363573	8.279295	-2.627748
29	O	-4.167340	7.846117	-2.622907
30	O	-4.808391	3.068868	-2.094048
31	O	-2.309971	-0.121601	-2.418753
32	O	2.263408	1.400994	-2.376602
33	O	2.674403	5.718485	1.091250
34	O	-0.116144	7.988616	0.581232
35	O	-3.755022	8.070749	0.801054
36	O	-4.970698	3.275525	0.968741
37	O	-2.357375	0.455297	0.796705
38	O	1.843249	1.001784	0.942907
39	O	-1.856821	2.270781	-2.180078
40	O	0.350837	4.271955	2.004265
41	O	0.824489	6.563372	3.239468
42	O	-2.111654	7.821762	3.029585
43	O	-4.116004	5.242093	3.114936
44	O	-3.514003	1.807157	3.243459
45	O	-0.266338	1.597529	2.948241
46	O	2.365459	3.382486	3.036492
47	O	1.396711	6.844994	-4.550589
48	O	-2.165402	7.002048	-4.591711
49	O	-4.735599	5.122439	-4.480101
50	O	-3.410762	2.037571	-4.535236
51	O	-0.277922	1.432041	-4.309354
52	O	1.789967	3.853887	-4.440857
53	O	-0.106639	5.104937	-0.611355
54	O	-1.682259	5.923543	1.532286

55	O	-4.209084	5.753029	0.517057
56	O	-2.369688	3.426526	1.916387
57	O	-0.519902	2.367054	0.390985
58	O	1.896675	3.301184	0.076418
59	O	-0.228112	5.517557	-3.220952
60	O	-2.596728	4.578008	-3.298258
61	C	1.458219	-6.453134	1.693782
62	C	1.956589	-6.687187	3.104682
63	N	2.458827	-7.822079	3.554659
64	C	2.628115	-8.919914	2.833299
65	C	2.274475	-9.059040	1.377844
66	S	1.527286	-7.738563	0.526645
67	C	3.186972	-10.136540	3.294126
68	C	3.373563	-11.145373	2.560012
69	C	3.099032	-11.362723	1.187888
70	C	2.495850	-10.203188	0.662562
71	C	0.846030	-4.108454	2.074652
72	C	1.334496	-4.424067	3.367647
73	C	1.805150	-5.491566	3.849064
74	C	0.953894	-5.257241	1.272738
75	N	0.365789	-2.915043	1.684516
76	N	3.356215	-12.489086	0.499937
77	C	4.068709	-13.603124	1.078302
78	C	3.131922	-12.584740	-0.924392
79	H	5.010864	-13.724851	0.505320
80	H	4.266706	-13.421166	2.135719
81	H	3.483707	-14.520145	0.941456
82	H	2.514807	-13.465584	-1.135011

83	H	4.119607	-12.749004	-1.401185
84	H	2.659535	-11.670684	-1.287054
85	C	-0.141497	-2.701761	0.342425
86	H	-0.018015	-3.617539	-0.238970
87	H	0.398808	-1.871461	-0.123316
88	H	-1.203600	-2.431208	0.396098
89	C	0.303889	-1.773531	2.574326
90	H	-0.745762	-1.506092	2.750997
91	H	0.803598	-0.920832	2.101863
92	H	0.782500	-2.013655	3.525908
93	Cl	6.294593	-13.627763	-1.469039

Table 1S. Final Coordinates (Angstroms) of each one of models analyzed in this work.
